# Design and Implementation of a Brief Digital Mindfulness and Compassion Training App for Health Care Professionals: Cluster Randomized Controlled Trial

**DOI:** 10.2196/49467

**Published:** 2024-01-22

**Authors:** Satish Jaiswal, Suzanna R Purpura, James K Manchanda, Jason Nan, Nihal Azeez, Dhakshin Ramanathan, Jyoti Mishra

**Affiliations:** 1 Department of Psychiatry University of California San Diego San Diego, CA United States; 2 Department of Mental Health, Veterans Affairs San Diego Medical Center San Diego, CA United States

**Keywords:** compassion, digital app, digital health, digital intervention, digital mental health, digital mindfulness, EEG, health workers, healthcare professionals, mindfulness, neuroplasticity, physicians, training

## Abstract

**Background:**

Several studies show that intense work schedules make health care professionals particularly vulnerable to emotional exhaustion and burnout.

**Objective:**

In this scenario, promoting self-compassion and mindfulness may be beneficial for well-being. Notably, scalable, digital app–based methods may have the potential to enhance self-compassion and mindfulness in health care professionals.

**Methods:**

In this study, we designed and implemented a scalable, digital app–based, brief mindfulness and compassion training program called “WellMind” for health care professionals. A total of 22 adult participants completed up to 60 sessions of WellMind training, 5-10 minutes in duration each, over 3 months. Participants completed behavioral assessments measuring self-compassion and mindfulness at baseline (preintervention), 3 months (postintervention), and 6 months (follow-up). In order to control for practice effects on the repeat assessments and calculate effect sizes, we also studied a no-contact control group of 21 health care professionals who only completed the repeated assessments but were not provided any training. Additionally, we evaluated pre- and postintervention neural activity in core brain networks using electroencephalography source imaging as an objective neurophysiological training outcome.

**Results:**

Findings showed a post- versus preintervention increase in self-compassion (Cohen *d*=0.57; *P*=.007) and state-mindfulness (*d*=0.52; *P*=.02) only in the WellMind training group, with improvements in self-compassion sustained at follow-up (*d*=0.8; *P*=.01). Additionally, WellMind training durations correlated with the magnitude of improvement in self-compassion across human participants (ρ=0.52; *P*=.01). Training-related neurophysiological results revealed plasticity specific to the default mode network (DMN) that is implicated in mind-wandering and rumination, with DMN network suppression selectively observed at the postintervention time point in the WellMind group (*d*=–0.87; *P*=.03). We also found that improvement in self-compassion was directly related to the extent of DMN suppression (ρ=–0.368; *P*=.04).

**Conclusions:**

Overall, promising behavioral and neurophysiological findings from this first study demonstrate the benefits of brief digital mindfulness and compassion training for health care professionals and compel the scale-up of the digital intervention.

**Trial Registration:**

Trial Registration: International Standard Randomized Controlled Trial Number Registry ISRCTN94766568, https://www.isrctn.com/ISRCTN94766568

## Introduction

Health care professionals receive intensive hands-on education and training so they may serve as resilient healers. Yet, with high workloads and multitasking demands, physicians can become vulnerable to workplace stress while making critical life-altering decisions for patients [1,2]. Evidence shows high rates of physician burnout (“workplace stress that has not been successfully managed,” *International Classification of Diseases*, World Health Organization), currently estimated at 44%, which is much higher than burnout in other professions [3,4]. Such chronic stress among physicians, in turn, may reduce the quality of physician-patient interactions and can lead to unprofessional behavior and attitudes [5,6]. Consistent with this, a study showed that burnout was associated with self-reported unprofessional conduct and less altruistic professional values among medical students at 7 US schools [7]. The COVID-19 pandemic also exacerbated distress in health care workers and has been shown to be associated with significant emotional pain, drop out from training, and reductions in work hours [8,9], which eventually affect the entire health care system. Hence, there is an emerging need for interventions that can help alleviate physician stress and prevent burnout [10].

Previous research has shown that once burnout develops, both individual-level and organizational strategies can result in clinically meaningful reductions in burnout [11], but organizational-level interventions (reducing work hours, increasing staffing, etc) have a larger overall effect size. [12] Notably, research has suggested that individual-level characteristics, particularly those related to mindfulness and self-compassion, may help to prevent burnout from developing [13-15]. Importantly, these are skills that can be developed. Research shows that when mindfulness and interoceptive-awareness exercises are provided as part of continuing medical education to primary care physicians, they help to reduce physician work-related stress and enhance well-being [11,16]. In a recent qualitative study among radio-oncologists, higher-trait mindfulness was found to be a protective factor from burnout and positively associated with life fulfillment [17]. Similarly, studies suggest that compassion training can promote well-being in medical students and improve the quality of clinical care [18]. Research has also shown that quality of care, health care costs, and the well-being of the clinician workforce are interlinked domains [19]. Hence, feasible, scalable, and effective mindfulness strategies delivered early may have far-reaching socioeconomic benefits for the health care system.

Compared to classroom approaches, digital training approaches can be highly scalable. Participants can flexibly engage with these trainings as per their convenience, so it is not a burden on busy work schedules [20]. Further, without the need for a teacher guide for digital training, the motivation for the practice is intrinsic and not reliant on teacher expertise. There are very few empirical studies among health care professionals that examine the efficacy of an app-based mindfulness program and its objective neural implications. A recent study in physician assistants showed a decrease in sleep dysfunction, enhanced connectivity between the medial prefrontal cortex and the superior temporal gyrus, as well as between regions critical for working memory after 8 weeks of intervention [21].

We have previously tested digital meditation approaches in adolescents and healthy young adults and shown positive neuro-cognitive outcomes [22,23]. Moreover, these digital trainings have integrated gamification, feedback, and rewards within closed-loop design systems to drive high user adherence [24].

In this study, we implemented a brief digital closed-loop training for health care professionals that involved an attention-to-breath practice with integrated compassion prompts. Respecting the time constraints of health professionals, each session provided 5-10 minutes of practice, and trainees had access to up to 60 digital sessions. Relative to a no-contact or business-as-usual control group, we evaluated the primary outcomes of change in mindfulness and self-compassion and monitored sessions of training engagement. While we additionally measured burnout, we did not expect this outcome to change with individual-focused training because it has been shown that organization-directed workplace interventions are more effective at addressing burnout [1,25] and burnout was also observed to be low in our sample. Thus, our main goal in this study was to examine whether mindfulness and compassion can be enhanced by brief digitally delivered practice sessions for health care professionals. Positive results from such a study may then serve as a rationale for future implementation integrated within an organizational framework to prevent burnout from developing.

Finally, this study also uniquely assayed objective neurophysiological plasticity associated with the training alongside subjective behavioral changes. For this, we measured electroencephalography (EEG)-based brain signals on an interoceptive attention-to-breathing task, assessed before and after training. The rationale for selecting such a task for neurophysiological measurements is that interoceptive attention to breathing is a core feature of several meditation practices [26,27]. On this task, we were particularly interested in neurophysiological activity within the default mode network (DMN), which has been shown to be modulated by meditation [28-31]. The DMN is a functional network that has not only been consistently associated with autobiographical memory and self-referencing but also on-task behavioral variability, mind-wandering, and rumination [32-37]. We hypothesized that mindfulness and compassion training would suppress DMN activity. Our ultimate analyses focused on whether objective modulation of the DMN relates to subjective behavioral changes in self-compassion and mindfulness.

## Methods

### Participants

A total of 43 human participants recruited in the study (mean age 28.77, SD 4.13; range 23-43 y; 20 male participants). All human participants were fluent in English. Participants were recruited from the University of California San Diego (UCSD) School of Medicine from Spring 2021 to Fall 2022 academic quarters through email advertisements and campus flyers.

Participants provided demographic data with regards to age, gender, and ethnicity. All participants were healthy adults, that is, they did not have any current medical diagnosis nor were taking any current psychotropic medications. Healthy status and affiliation to the UCSD School of Medicine were the only eligibility criteria.

Participants completed the Maslach Burnout Inventory (MBI) at the time of screening; MBI scores did not reflect high burnout in our sample as all scores were less than the midscore of the MBI score range (see the *Results* section).

### Study Design

The study design was interventional and cluster randomized. Of the total 43 study participants, 22 were enrolled in the digital WellMind intervention group, and 21 were part of the no-contact control group. Participants were cluster randomized based on the academic quarter of enrollment to the WellMind or control group. Specifically, all participants recruited during Spring 2021, Spring 2022, and Fall 2022 academic quarters were assigned to the WellMind group, and the no-contact control group participants were recruited during Fall 2021. This was done because individuals within each academic quarter (but not across quarters) were working or studying together and, hence, knew each other professionally and could reveal components of the study intervention to each other. The WellMind group participants received the digital app intervention and had periodic email contact from our research team during the intervention, at about once every 2 weeks, to ensure compliance and help troubleshoot any issues faced by the participants. On the other hand, the no-contact control group had no interaction with the study research team or any digital training resource provided to them between their pre- and postintervention time points.

### Sample Size and Power

The sample size within each group was powered to detect medium effect size for pre- or postintervention differences (Cohen *d*>0.6) at β power of 0.8 and α level of 0.05. Between-group differences met criteria for investigating only large effect size outcomes (Cohen *d*>0.8) at β power of 0.8 and α level of 0.05. Effect sizes were calculated a priori using the G*Power (Axel Buchner) software [38].

### Intervention

The WellMind digital intervention was deployed on the BrainE platform, implemented in Unity Game Engine (Unity Technologies), and available on both iOS and Android phone devices [39]. This digital program is Health Insurance Portability and Accountability Act (HIPAA) compliant and secured by password protection, and each user interacts through an alphanumeric study ID that is not linked to any personal health information. Participants accessed the app in their own free time and engaged in breath-focused mindfulness training, with each session lasting 5-10 minutes for up to 60 sessions. The training was delivered in a game-like format and was performance adaptive. Specifically, individuals were requested to close their eyes, pay attention to their breathing, and tap the mobile screen after a specific number of breaths. The app monitored the consistency of tap responses. If the user was distracted based on the low consistency of breath monitoring taps, a gentle chime reminded the user to let go of the distraction and revert their attention back to mindful breathing. Initially, at level 1, participants tapped the screen after each breath. If they were able to do this consistently for 3 repeats of level 1 of 1 minute duration each, they graduated to level 2 and tracked 2 breaths at a time for 2 minutes, and so on. Thus, in the performance-adaptive task, the level reflected the number of minutes spent at that level and the number of breaths the participant was requested to repeatedly monitor. The maximum achievable level was level 10, that is, monitoring 10 breaths at a time for up to 10 minutes. When the user graduated to the max level, they stayed at this level until the end of all assigned sessions, that is, 60 sessions. Also within the game-like format, when the participant opened their eyes at the end of a level, a peaceful nature scene would slowly unfold as a form of training reward.

Overall, this digital meditative practice is considered closed loop because of its performance-adaptive feature [22,23]. Consistent attention to breathing is emphasized over other types of breathing techniques, such as deep breathing. The moment-to-moment performance tracking further allows quantification of the attentive focus during each session, which is not possible with traditional nondigital meditation.

**Figure 1:**
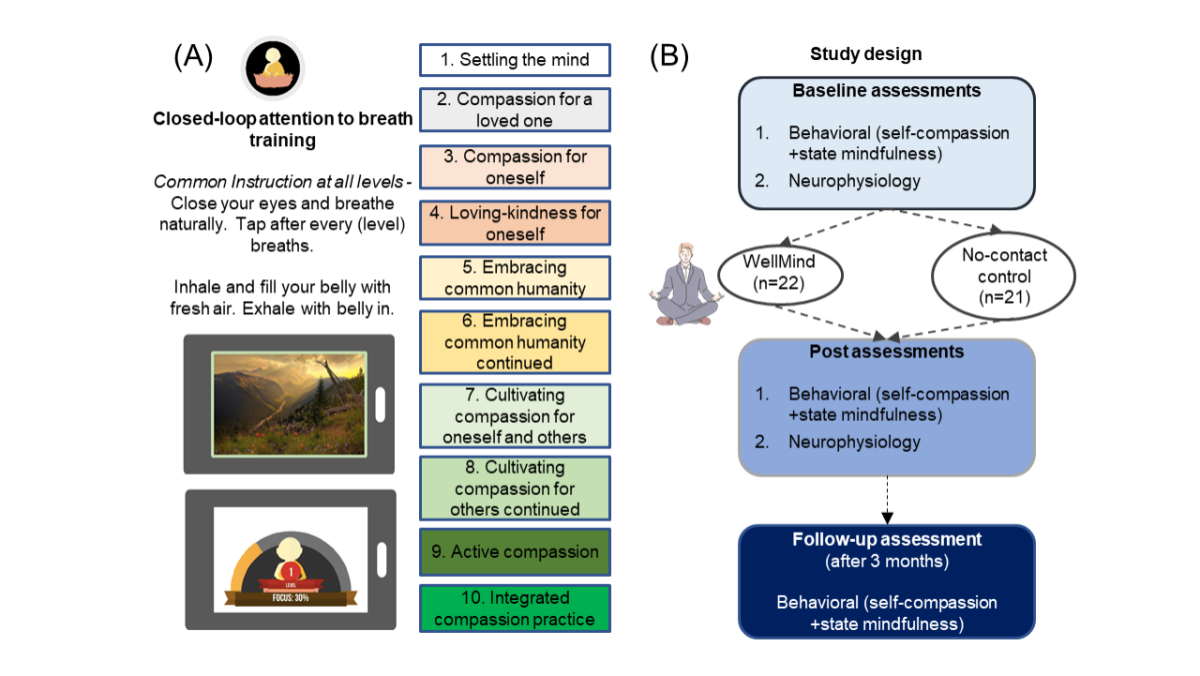
Brief digital mindfulness and compassion training. (A) The WellMind app delivered closed-loop, that is, performance-adaptive, attention to breath training. Common instruction across 10 levels of training is shown. At each level, the user tapped the mobile screen after (certain level) number of breaths while keeping their eyes closed. Feedback included auditory chimes to guide consistent performance and signal the end of training. A distinct, calming nature scene is unveiled at the end of each block in the session, along with focus feedback based on consistency of performance. Levels of breath monitoring were tied to 10 levels of cultivating compassion instructions. (B) The study design incorporated behavioral and neurophysiological assessments at pre- and posttraining and continued behavioral assessments at follow-up conducted in the WellMind training group relative to a no-contact control group.

Finally, the training also introduced standard compassion cultivation instructions as audio and text prompts before the start of each session’s breath practice. Prompts were updated every 6 sessions, with a total of 10 prompts gradually increasing in complexity over 60 sessions. These prompts were designed per guidance from the Compassion Cultivation Training program [40] and included (1) settling the mind, (2) compassion for a loved one, (3) compassion for oneself, (4) loving kindness for oneself, (5) embracing common humanity, (6) embracing common humanity continued, (7) cultivating compassion for oneself and others, (8) cultivating compassion for others continued, (9) active compassion, and (10) integrated compassion cultivation practice. The WellMind training app and study design are summarized in Figure 1. Participants received in-app notifications once a day, reminding them to complete their training.

### Behavioral Assessments

At baseline (T1), postintervention completion (T2; or a 3-month no-contact period for the control group), and at follow-up (T3; 6 months following baseline), participants completed validated behavioral self-report scales of self-compassion: a 12-item self-compassion scale [41], and mindfulness: a 14-item Mindful Attention Awareness Scale [42]. These measures served as the primary outcomes. MBI measures were obtained as exploratory outcomes at T1 and T2. The Cronbach α measure of reliability was calculated for each of these behavioral measures at baseline.

### Neurocognitive Assessments

In addition, participants completed an objective neurophysiological assessment of interoceptive attention to breathing at T1 and T2. For these assessments, all participants made individual study visits at the Neural Engineering and Translational Labs at the UCSD. Assessments were deployed on the BrainE platform with simultaneous EEG [43], delivered on a laptop (running on the Windows 10 operating system) at a comfortable viewing distance. The Lab Streaming Layer protocol was used to time stamp all user response events in this assessment [44].

In the interoceptive attention to breathing task, participants were instructed to close their eyes, breathe naturally, and respond every 2 breaths by tapping on the spacebar [45,46]. The computer screen appeared gray for the 5-minute duration of the task, implemented in two 2.5-minute blocks. A beep signaled the end of the task, at which time participants opened their eyes. The median response time (RT) on the interoceptive task was monitored for all human participants so that we could identify and contrast neurophysiological activity on high consistency, that is, attentive breath monitoring trials (trials with RT≤1 median absolute deviation of median RT) versus low consistency, that is, distracted trials (trials with RT>1 median absolute deviation of median RT) in each human participant.

EEG data were collected using a 24-channel cap with saline soaked electrodes following the 10-20 system and a wireless SMARTING amplifier (mBrainTrain). The signals were digitized with a sampling rate of 500 Hz and 24-bit resolution and stored as .xdf files.

### Behavioral Data Analyses

For behavioral subjective scales, scores on self-compassion and mindfulness were calculated at T1, T2, and T3 and for MBI at T1 and T2. T2 versus T1 and T3 versus T1 scores were compared within each group using 2-tailed paired *t* tests or its nonparametric equivalent Wilcoxon signed rank test depending on the distribution of the behavioral scores; the normality of distributions was checked using the Levene test. For mindfulness, we compared state mindfulness across the 3 time points, which is a component of the dispositional trait mindfulness scale [47], as we expected state mindfulness but not trait mindfulness to be malleable with training.

Cohen *d* effect sizes were calculated for both within and between-group differences. Repeated measures analyses of variance comparing between-group behavioral differences were not conducted in this first study given the large effect sizes (*d*>0.8) needed to observe significant group differences with adequate power.

To investigate the relationship between outcome gains and training engagement, behavioral changes in the WellMind training group were correlated with the number of training sessions completed by participants using Spearman correlations.

### Neurocognitive Data Analyses

We applied a uniform processing pipeline to all EEG data published in several of our studies [43,45,46,48-53]. This included (1) EEG channel data processing and (2) cortical source localization of the EEG data to estimate source-level neural activity. Details of this analysis are provided in the Multimedia Appendix 1 [51,53-68].

Alpha band EEG data were trial averaged for high versus low consistency (ie, attended vs distracted) breath monitoring trials on the interoceptive attention task. These trials were compared for within-group pre- versus postintervention activity differences in the fronto-parietal network (FPN), cingulo-opercular network (CON), and DMN using paired *t* tests. Effect sizes were also calculated for neural data, reported as Cohen *d*: 0.2=small, 0.5=medium, and 0.8=large [69]. Given that we have observed large effect size neural outcomes (*d*>0.8) in our previous digital training studies [22,23,70], repeated measures ANOVA was conducted to analyze between-group post- versus preintervention network effects; the Greenhouse-Geisser significance correction was applied to adjust for lack of sphericity. Finally, Spearman correlations were used to analyze neurobehavioral associations.

### Ethical Considerations

Each participant gave written informed consent in accordance with the Declaration of Helsinki before participating in the experiment. All the experimental procedures were approved by the institutional review board of the University of California San Diego (protocol #180140). All data is de-identified, and up to US $150 in compensation was provided as an e-Gift card to all participants for completing all aspects of the study, including assessments and intervention procedures.

## Results

### Baseline Group Comparisons

There were no demographic differences between the 2 groups for age, gender, and ethnicity (Table 1). Age comparisons were made using the Wilcoxon sum rank test, and gender and ethnicity comparisons were made using chi-square tests.

**Table 1:** Summary of participant demographics and baseline behaviors. Data were checked for normality, and WellMind and control groups were appropriately compared using the *t* test if normal, or else using the nonparametric Wilcoxon sum rank test. Gender and ethnicity variables were compared using chi-square tests.

Demographics and baseline behaviors			WellMind (n=22)		Control (n=21)		Group difference *P* value
Age (years), mean (SD)			27.91 (3.15)		29.67 (4.96)		.32
**Gender n (%)**							.55
	Men	8 (36)		12 (57)			
	Women	14 (64)		8 (38)			
**Ethnicity n (%)**							.19
	Asian	8 (36)		8 (38)			
	Black or African American	0 (0)		1 (5)			
	American Indian or Alaska Native	0 (0)		0 (0)			
	White	10 (46)		9 (43)			
	More than 1 ethnicity	3 (14)		2 (10)			
	Other	1 (5)		1 (5)			
Trait mindfulness, mean (SD)			3.18 (0.66)		3.14 (0.77)		.84
Self-compassion, mean (SD)			2.69 (0.57)		3.09 (0.73)		.05
MBI^a^ emotional exhaustion, mean (SD)			16.27 (4.45)		24.05 (10.22)		.02^b^
MBI personal accomplishment, mean (SD)			21.41 (3.63)		32.86 (6.73)		<.001^c^
MBI depersonalization, mean (SD)			5.36 (3.33)		9.29 (7.93)		.23

^a^MBI: Maslach Burnout Inventory.

^b^*P*<.05.

^c^*P*<.001.

Our main outcome variables of mindfulness and self-compassion also did not show significant group differences at baseline. Burnout measures on the MBI showed significantly less emotional exhaustion and sense of personal accomplishment in the WellMind group relative to the control group at baseline, but no differences in MBI depersonalization. Overall, burnout levels in the WellMind group were low, that is, less than a scale midscore of 18 for emotional exhaustion, greater than a scale midscore of 16 for sense of personal accomplishment, and less than a scale midscore of 10 for depersonalization.

All behavioral measures had consistently high Cronbach α measured across all human participants at baseline (mindfulness: α=.81, self-compassion: α=.86, and MBI: α=.88).

### Behavioral Results

**Figure 2:**
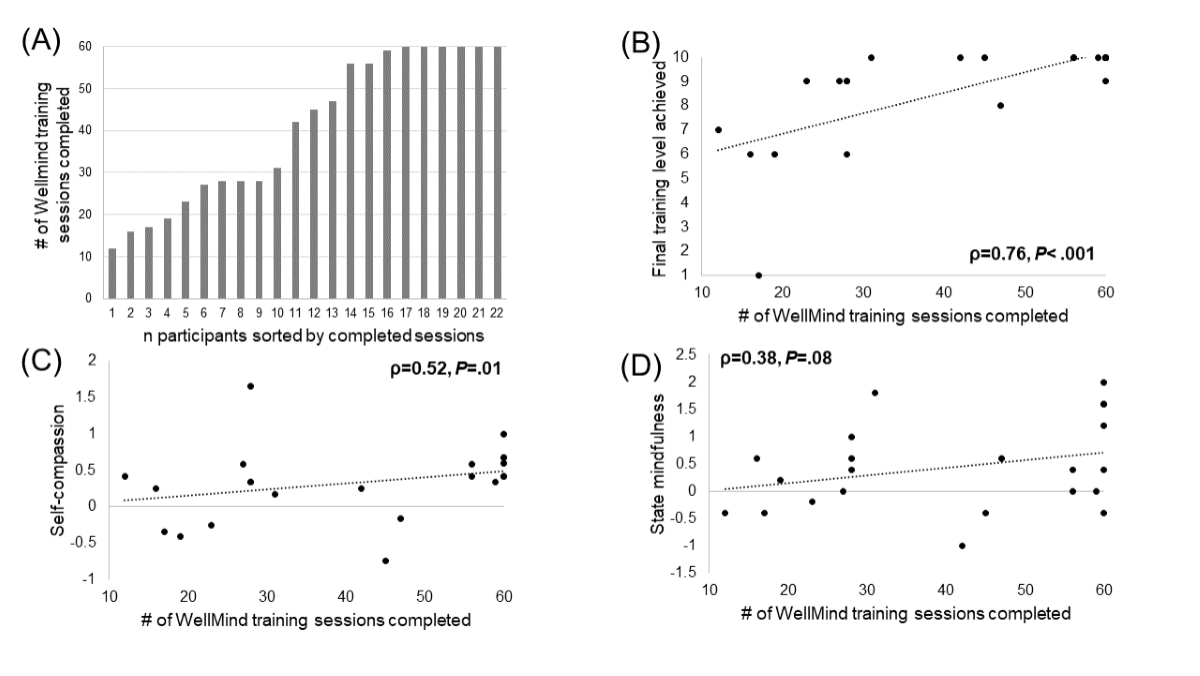
WellMind training sessions completed and relationship with outcomes. (A) The number of training sessions completed by each WellMind participant. (B) The final level of breath training is significantly related to the number of completed training sessions. (C) Post- versus prechange in self-compassion is significantly related to number of completed training sessions. (D) Post- versus prechange in state mindfulness showed a trend in its relationship to the number of completed training sessions. The Spearman correlation results are shown.

Participants in the WellMind group completed 40.64 (SD 17.79) training sessions on average, or 68% (40.64/60) of the total 60 sessions; the number of training sessions completed by different participants is shown in Figure 2A. The number of training sessions completed directly related to the final level achieved in training across participants (ρ=0.76, *P*<.001; Figure 2B). Additionally, we found that the number of training sessions was also significantly related to post- versus preintervention increase in self-compassion (ρ=0.52, *P*=.01; Figure 2C) and trended toward significance in relation to post- versus preintervention increase in mindfulness (ρ=0.38, *P*=.08; Figure 2D), but this was not statistically significant.

**Figure 3:**
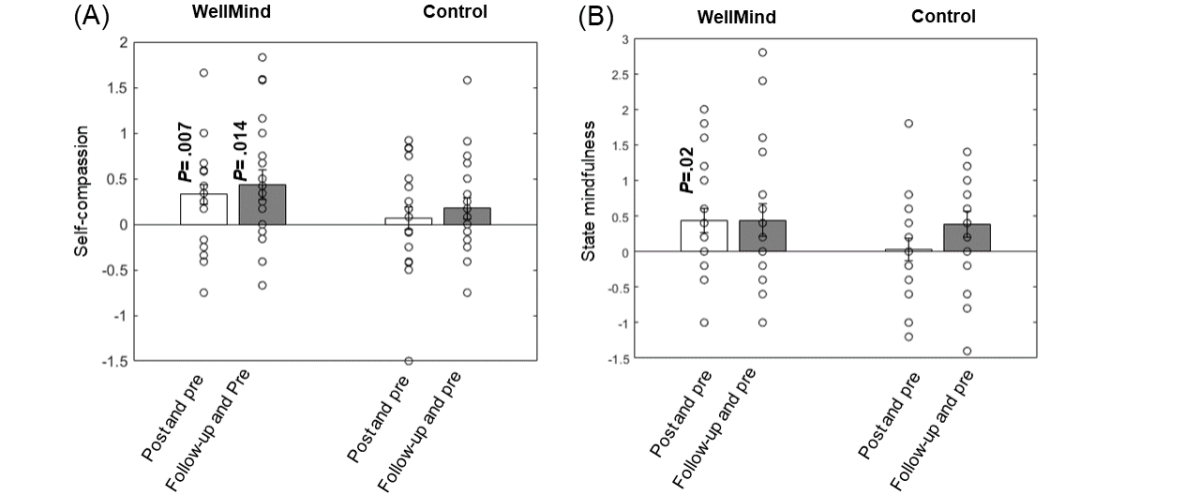
Post- versus preintervention (T2 vs T1) and follow-up versus preintervention (T3 vs T1) self-compassion and state mindfulness outcomes in the WellMind (n=22) and control (n=21) group; data at follow-up were missing for 2 participants in the WellMind group and for 1 participant in the control group. Both (A) self-compassion and (B) state mindfulness scores significantly improved in the WellMind group. Bar plots show the change in score mean and standard error about the mean for error bars, with the actual distribution of scores shown as scatter points. As self-compassion and state mindfulness measures had normal distributions, *P value results are from paired t tests between pre- and postintervention or preintervention and follow-up assessments.

The within-group changes in primary measures of self-compassion and state mindfulness and exploratory measures of burnout (MBI scores) between post- versus preintervention and follow-up versus preintervention sessions are shown in Tables 2 and 3 for the WellMind and control groups. A significant increase in self-compassion and mindfulness was observed at post- versus preintervention sessions only in the WellMind group, and the significant increase in self-compassion was sustained at follow-up (Figure 3). As the self-compassion scale has 2 components for compassionate self-responding (CSR) and uncompassionate self-responding (USR) [71], we also analyzed whether either CSR or USR undergo significant training-related change. CSR reflects self-kindness, common humanity, and mindfulness, while USR reflects the opposite constructs of self-judgment, isolation, and overidentification. We found that the WellMind training-related improvement in self-compassion was exclusively driven by a reduction in USR (post vs preintervention change: –0.42, SD 0.55; *P*=.002; and follow-up vs preintervention change: –0.82, SD 1.27; *P*=.009), but there was no significant change in CSR at post (*P*=.17) or follow-up (*P*=.07). Baseline burnout in our sample was low, and there were no significant post- versus preintervention changes in MBI scores in both groups.

Cohen *d* effect sizes for within-group differences were calculated as the mean difference between post- versus preintervention (or follow-up vs preintervention) outcomes expressed in pooled SD units. Cohen *d* effect sizes were also calculated for between-group differences. Effect sizes for significant self-compassion and mindfulness outcomes were in the medium range (Tables 2 and 3).

**Table 2:** Summary of behavioral outcomes obtained at baseline (T1) and 3 months (postintervention; T2 after baseline. The primary outcome measures for the study, self-compassion and state mindfulness, showed significant changes in the WellMind group at postintervention time point and are noted. The control group did not show any significant changes. Data were checked for normality, and pre- versus postintervention within-group differences were appropriately compared using a paired *t* test if normal, or else using the nonparametric Wilcoxon sign rank test. Both within-group and between-group Cohen *d* effect sizes were calculated.

Pre- versus postintervention outcomes		WellMind					Control					Between-group effect size
		T1, mean (SD)	T2, mean (SD)	Effect size	Group difference *P* value	T1, mean (SD)		T2, mean (SD)	Effect size	Group difference *P* value		
Self-compassion		2.69 (0.57)	3.02 (0.58)	0.57	.007^a^	3.09 (0.73)		3.15 (0.67)	0.09	.61	0.49	
**State mindfulness**		2.94 (0.79)	3.37 (0.85)	0.52	.02^b^	3.22 (0.94)		3.25 (0.82)	0.03	.86	0.53	
	MBI^c^ emotional exhaustion	16.27 (4.45)	15 (5.86)	–0.24	.15	24.05 (10.22)		23.05 (12.45)	–0.09	.89	–0.04	
	MBI personal accomplishment	21.41 (3.63)	21.64 (4.41)	0.06	.61	32.86 (6.73)		34.67 (7.77)	0.25	.14	–0.35	
	MBI depersonalization	5.36 (3.33)	4.5 (3.71)	–0.24	.23	9.29 (7.93)		7.57 (7.37)	–0.22	.39	0.18	

^a^*P*<.001.

^b^*P*<.05.

^c^MBI: Maslach Burnout Inventory.

**Table 3:** Summary of behavioral outcomes obtained at baseline (T1) and 6 months after baseline (follow-up; T3). Data were checked for normality, and pre- versus postintervention within-group differences were appropriately compared using a paired *t* test if normal, or else using the nonparametric Wilcoxon sign rank test. Both within-group and between-group Cohen *d* effect sizes were calculated.

Pre- versus postintervention outcomes	WellMind					Control					Between-group effect size
	T1, mean (SD)	T3, mean (SD)	Effect size	Group difference *P* value	T1, mean (SD)		T3, mean (SD)	Effect size	Group difference *P* value		
Self-compassion	2.69 (0.57)	3.14 (0.55)	0.80	.014^a^	3.09 (0.73)		3.29 (0.54)	0.31	.16	0.41	
State mindfulness	2.94 (0.79)	3.41 (1.08)	0.50	.07	3.22 (0.94)		3.55 (0.78)	0.38	.05	0.06	

^a^*P*<.001.

### Neurocognitive Results

**Figure 4:**
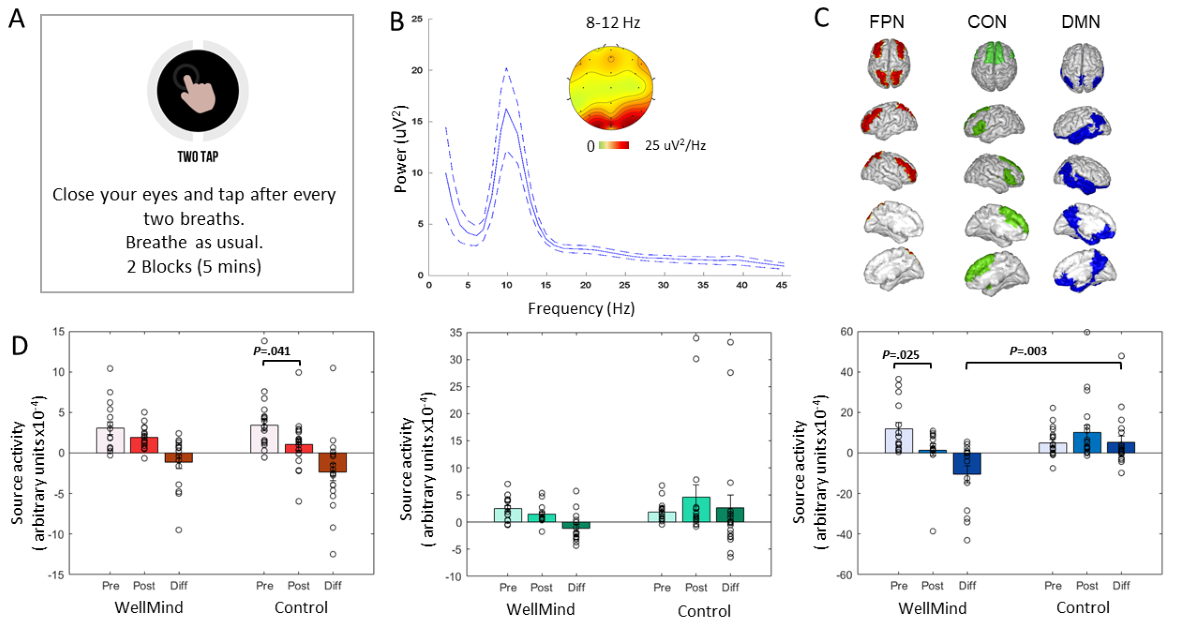
Training-related neurophysiological changes evaluated on the attention-to-breath monitoring assessment. (A) Schematic of task instructions. (B) A power frequency plot of scalp channel data across all participants and sessions showed peak processing in the α frequency band (8-12 Hz). (C) Source-reconstructed electroencephalography data were analyzed for 3 networks: frontoparietal network (FPN), the cingulo-opercular network (CON), and the default mode network (DMN); regions of interest averaged within each network are shown. (D) Comparisons of the WellMind versus control group network activity showed significant reduction in activity only for the WellMind group in the DMN; bar plots show mean and standard error about the mean α band activity (y-axes: 10–4 cortical source activity arbitrary units) within the 0- to 4-second epoch before breath responses, as well as the relative response on low versus high consistency (ie, distracted versus attended) trials at pre- and postintervention time points and the post-pre difference. Actual activity distributions are shown as scatter points.

We analyzed post- versus preintervention modulation of source-localized neural activity on the interoceptive attention to breathing assessment in brain regions of interest collated within canonical cognitive control networks, specifically the FPN, CON, and DMN. There were no between-group differences in neural network activity at baseline (*P*>.05). The within-group changes in network activity between post- versus preintervention sessions are shown in Table 4 for the WellMind and control groups. Per our hypothesis, a significant decrease in activity in the mind-wandering and rumination-associated DMN was observed at post versus pre sessions only in the WellMind group (Figure 4). Besides this, the only other significant change observed was a decrease in post- versus preintervention FPN activity in the control group.

Cohen *d* effect sizes were calculated for both within-group and between-group differences (Table 4). Notably, the reduction in post- versus preintervention DMN activity in the WellMind group showed both large within-group and between-group effect sizes (DMN between group repeated measures ANOVA: session interaction: *F*_1_,_32_=10.11; *P*=.003; η^2^=0.24; main effects of group or session were not significant, *P*>.30; also, the other networks, FPN and CON, did not show any significant between-group effects, *P*>.10).

**Table 4:** Summary of neural activity outcomes obtained at baseline (T1) and at 3 months (postintervention; T2). The primary network of interest for the study, the default mode network (DMN), showed significant change in the WellMind group at post- versus preintervention and is noted. Besides, the control group showed a significant change in fronto-parietal network (FPN) activity. Mean (SD) data are shown in 10-4 cortical source arbitrary units for all variables. Post- versus preintervention within-group and between-group Cohen *d* effect sizes are calculated.

Pre- versus postintervention outcomes	WellMind					Control					Between-group effect size
	T1, mean (SD)	T2, mean (SD)	Effect size	Group difference *P* value	T1, mean (SD)		T2, mean (SD)	Effect size	Group difference *P* value		
FPN	3.08 (3.14)	1.96 (1.45)	–0.46	.21	3.45 (3.39)		1.08 (3.08)	–0.73	.04^a^	0.30	
CON^b^	2.55 (2.18)	1.44 (1.76)	–0.56	.14	1.88 (1.77)		4.57 (9.86)	0.38	.27	–0.48	
DMN	11.92 (12.55)	1.44 (11.66)	–0.87	.03^a^	4.94 (7.10)		10.26 (15.43)	0.44	.09	–1.10	

^a^*P*<.05.

^b^CON: cingulo-opercular network.

### Neurobehavioral Associations

**Figure 5:**
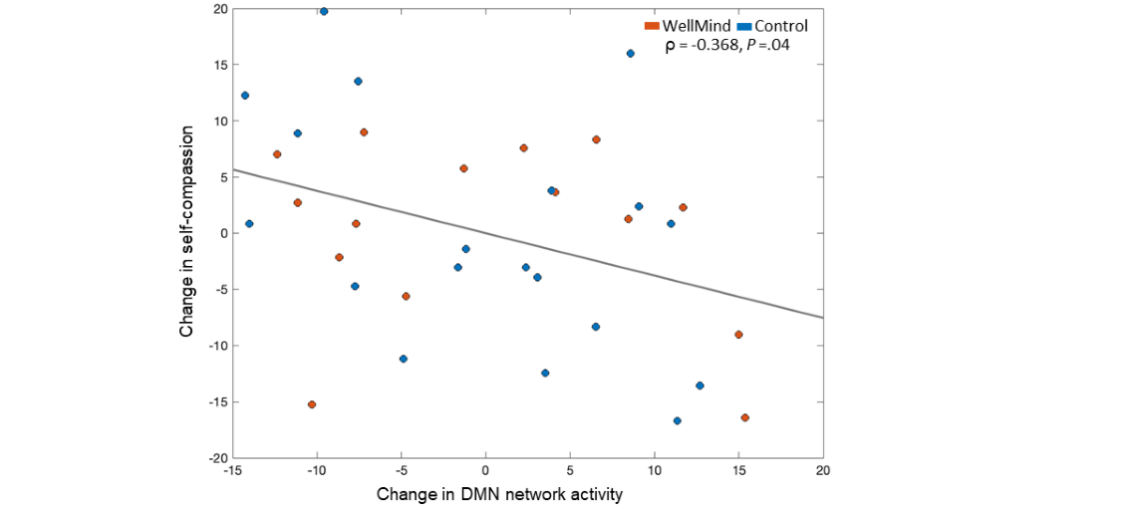
Relationship between post- versus preintervention change in self-compassion scores and change in default mode network (DMN) network activity. Residuals are plotted using Spearman partial correlation, taking into account group assignment to the WellMind or control group and baseline burnout score differences between groups.

We conducted neurobehavioral correlation analyses between neural measures of post- versus preintervention change in DMN activity and change in primary behavioral outcomes of self-compassion or state mindfulness. Spearman partial correlations were implemented that accounted for participant groups and their baseline burnout score differences (MBI; Table 1). We found a significant relationship between change in DMN network activity and change in self-compassion (ρ=–0.368; *P*=.04; Figure 5) but not state mindfulness (*P*>.50). Specifically, individuals who showed the largest improvement in self-compassion also showed the greatest DMN suppression. We also verified that these effects were driven by the relationship between DMN suppression and reduction in USR (ρ=0.37; *P*=.04), but there was no significant relationship with change in CSR (*P*=.35).

We also found that the extent of training-related DMN suppression in the WellMind group was significantly related to their baseline DMN activity (ρ=–0.74; *P*=.002); this result did not change when controlling for the number of WellMind training sessions completed, and no such relationship was observed for the control group (*P*=.71). These results suggest that baseline DMN activity could be a predictive marker for the extent of training-related neural plasticity in this network, which in turn relates to improvement in self-compassion.

## Discussion

In this study, we developed WellMind, a digital mindfulness and compassion training, and implemented it with health care professionals. Respecting their real-world time constraints, the training was brief (5-10 minutes per session) and available for up to 60 sessions. The novelty of the training lay in its closed-loop, that is, performance-adaptive mechanics and quantitative feedback design applied to attentive breathing, with levels of compassion cultivation instructions. To account for the practice effects of repeat assessments, we included a no-contact control group in the study design. We found that the WellMind intervention significantly improved the primary behavioral outcomes of self-compassion and mindfulness, with the improvements in self-compassion sustained at follow-up. No such behavioral effects were observed in the control group, and overall effect sizes were in the medium range. Concomitantly, we also found training-related neurophysiological suppression of the DMN, which is implicated in mind-wandering and rumination, and the extent of DMN suppression related to significant improvements in self-compassion.

A recent meta-analytic review of 27 studies [72] and randomized controlled trials found that smartphone apps can be used to enhance mindfulness and compassion skills, as well as reduce stress [73,74]. Additionally, a recent scoping review concluded that it is feasible to deliver compassionate care within digital health care, particularly telemedicine [75]. Yet, previous digital interventions in this field have reported small effect sizes [72]. In this study, we found medium effect size behavioral changes that further correlated with the extent of digital training completed across human participants, highlighting the advantages of this closed-loop digital training. It is also notable that the medium effect size improvements in self-compassion were sustained at follow-up; this finding is aligned with evidence from our past digital intervention studies showing robust long-term behavioral effects [22,76].

The core element of our digital WellMind training is the attention to breathing practice, which is also a foundational element of traditional meditation; indeed, meditation practice has been shown to enhance state-mindfulness [77,78]. Yet, traditional practice lacks real-time feedback and performance-based level progressions and can have variable outcomes depending on the teacher [79,80]. WellMind is a teacher-independent digital app within which user feedback is key to maintaining engagement over multiple sessions [24]. Notably, as users engaged with more sessions, we also observed them progress to higher levels of breath monitoring (maximum monitoring of 10 breaths at a time)—a significant correlation was found between breath monitoring level and training sessions completed. At higher training levels, progressively more sophisticated compassion instructions were also unveiled, which may be driving the correlation between the number of training sessions completed and improvements in self-compassion. Furthermore, when we investigated the positive (CSR) and negative (USR) subcomponents of the self-compassion scale [71], we found that reduction in USR (ie, reduction in self-judgment, isolation, and overidentification) exclusively underlies the training-related improvement in total self-compassion. To the best of our knowledge, previous research has not addressed the differential intervention-related plasticity of CSR versus USR components but has shown that these are distinct constructs that differentially relate to well-being and cognitive responses to daily life problems [81,82].

With regard to real-time feedback, WellMind integrates breath monitoring consistency-based auditory feedback as a gentle chime that signals the user to return to attentive breath monitoring when distracted. Indeed, auditory feedback during focused attention meditation has been shown to improve state mindfulness [83]. Notably, in large sample surveys of perceived barriers to meditation across hundreds of participants, a lack of individualized feedback and progress tracking have been prominently cited as important hindrances [84,85]. WellMind was designed to remove these barriers and, hence, motivate training. It is also distinct from previous closed-loop digital meditation approaches that prompt the user to retrospectively and subjectively report whether they were attentive or distracted during their meditation practice and did not integrate compassion cultivation [22,23].

In addition to behavioral outcomes, we also investigated EEG-based neurophysiological outcomes evaluated on an interoceptive breath monitoring task. The eyes-closed interoceptive task required monitoring of 2 breaths at a time and was devoid of feedback or performance-adaptive levels, and thus served as a pure assessment of breath-focused interoception that was targeted by the WellMind training. Consistent with our hypothesis based on previous evidence [28,29,31], we observed training-related suppression of cortical source-localized DMN activity, with no such changes found in the control group. The DMN outcome had a large between-group effect size. In contrast, the FPN and CON executive control networks did not show training-related changes; the FPN showed a significant within-group post versus pre activity reduction in the control group that could be related to attentional lapses or boredom during repeat assessments in this group [22,86,87]. Finally, neurobehavioral correlations showed that training-related DMN activity suppression was significantly related to improvement in self-compassion, specifically reduction in the USR component. Also, individuals with high DMN activity at baseline experienced greater DMN suppression with training, suggesting baseline DMN activity as an individual-specific neural marker to determine who may benefit most from such training. Overall, it is worth noting that we obtained these results using EEG, a scalable approach for measuring neural markers relative to other neuroimaging modalities such as functional magnetic resonance imaging. We also note that in our previous work, we have shown high test-retest reliability of such EEG data collected within the context of cognitive tasks [43].

As per the limitations of this study, the sample size and nonactive control group are the primary limitations, although it is worth noting that this is the first ever implementation of the digital WellMind intervention in health care professionals. We also acknowledge other limitations of our intervention design, such as that participants were not blind to the intervention condition and that WellMind participants received a greater frequency of contact from the experimenters during the intervention (email reminders to encourage adherence), while the control group had no such contact. These differences may lead to differences in expectations, resulting in positive outcomes observed in the WellMind group. Also, burnout in our sample was low, and we did not observe any post versus pre changes in burnout. Yet, previous work has suggested that organization-directed workplace interventions could be more effective at addressing burnout [25] and a recent study showed that physical exercise facilitated by mobile app technology also benefits burnout [88]. In this context, our research presents the possibility that individual interventions focused on self-compassion and mindfulness delivered before the development of burnout may be helpful in burnout prevention. Our data, showing long-term benefits in self-compassion with moderate to large effect sizes, suggests this approach may be useful in preventing burnout. Yet, this field has acknowledged that there are challenges to implementing behavioral interventions at the organizational level, especially in health care [8]. These challenges include a clear lack of time for physicians as well as concerns regarding confidentiality and discrimination within this workforce. In this context, WellMind offers brief training sessions (5-10 minutes in duration) that can be engaged flexibly at any time of day. Its closed-loop features promote adherence, although we acknowledge that not everyone is similarly motivated, as reflected by the variable number of sessions completed by the training participants. Security and confidentiality should be a paramount concern for any digital intervention [89,90]. Hence, WellMind access is enabled on a secure, password-protected HIPAA-compliant platform, with only deidentified data available for review and analyses. These features can facilitate future adoption. Overall, it is recommended that such interventions are embedded within the early stages of physician education as a foundational resource, when there may be more availability of time, to enhance receptivity, adherence, and build resilience to future burnout.

In conclusion, this study showcases an accessible and closed-loop approach to foster compassion and mindfulness among health care professionals. We found significant behavioral effects as well as robust brain plasticity related to these effects. These findings encourage future scale-up of the digital intervention to promote physician well-being and prevent burnout.

## Appendix

Multimedia Appendix 1 Supplementary methods.

Multimedia Appendix 2 CONSORT-EHEALTH checklist (V 1.6.1).

## Abbreviations

CON: cingulo-opercular network
CSR: compassionate self-responding
DMN: default mode network
EEG: electroencephalography
FPN: fronto-parietal network
HIPAA: Health Insurance Portability and Accountability Act
MBI: Maslach Burnout Inventory
RT: response time
UCSD: University of California San Diego
USR: uncompassionate self-responding

## Data Availability

The data set generated and analyzed in this study are available from the Data Dryad repository [91].

The WellMind digital intervention on the BrainE platform app is available for health care education and research on the website and is copyrighted for commercial use (Regents of the University of California Copyright #SD2024-132) [92].
