# Supplementary material for: Design and Implementation of a Brief Digital Mindfulness and Compassion Training App for Health Care Professionals: Cluster Randomized Controlled Trial
Source: JMIR Ment Health. 2024 Jan 22;11:e49467. doi: 10.2196/49467 (PMC10845023; doi:10.2196/49467)
Supplement: Multimedia Appendix 1 [file mental_v11i1e49467_app1.docx]

**Multimedia Appendix 1: Supplementary Methods**

**Neuro-cognitive (EEG) Data Analyses.**

This included: 1) EEG channel data processing, and 2) cortical source localization of the EEG data to estimate source-level neural activity.

1) EEG channel data processing was conducted using the EEGLAB toolbox v2020 in MATLAB v2020 [54]. EEG data was resampled at 250 Hz and filtered in the 1-45 Hz range to exclude ultraslow DC drifts at <1Hz and high-frequency noise produced by muscle movements and external electrical sources at >45Hz.

Within the interoceptive attention task, EEG data were average-referenced and epoched to the LSL time-stamps of the response taps made by participants after every two breaths. Trials were epoched in the -4.0 sec to +4.0 sec window around response and categorized as either high consistency, i.e. attentive trials (trials with RT $\leq$ 1 median absolute deviation of median RT) or low consistency, i.e. distracted trials (trials with RT > 1 median absolute deviation of median RT). This epoch was chosen because it was not contaminated by motor artifacts given that median response times for two-breath monitoring across subjects were ~8 sec in the interoceptive task.

There were no missing channels in the EEG data across subjects. Epoched data were cleaned using the autorej function in EEGLAB to remove noisy trials, i.e. >5SD outliers rejected over max 8 iterations, followed by further cleaning of electrooculographic, electromyographic or non-brain source artifacts using the Sparse Bayesian learning (SBL) algorithm (https://github.com/aojeda/PEB) [53,55].The cleaned data were then band filtered in the physiologically relevant theta (4-7 Hz), alpha (8-12 Hz), and beta (13-30 Hz) frequency bands. Given that alpha band oscillations are dominant during eyes closed [56-59], as was also evidenced in our data (see Results), we exclusively source localized alpha band neural processing.

2) We used the block-Sparse Bayesian learning (BSBL-2S) algorithm to localize the alpha frequency band filtered EEG data and partitioned the signals into cortical regions of interest (ROIs) and artifact sources [53,55]. BSBL-2S is a two-step algorithm in which the first-step is equivalent to low-resolution electromagnetic tomography (LORETA [60]). LORETA estimates sources subject to smoothness constraints, i.e. nearby sources tend to be co-activated, which may produce source estimates with a high number of false positives that are not biologically plausible. To guard against this, BSBL-2S applies sparsity constraints in the second step wherein blocks of irrelevant sources are pruned. Notably, this data-driven sparsity constraint reduces the effective number of sources considered at any given time as a solution, thereby reducing the uncertainty of the inverse solution. Thus, it is not that only higher channel density data can yield source solutions, the ill-posed inverse problem can also be solved by imposing more aggressive constraints on the solution to converge on the source model at lower channel densities, as also supported by prior research [61,62]. Of note, the BSBL-2S two-stage algorithm has been benchmarked to produce evidence-optimized inverse source models at 0.95AUC relative to the ground truth [53,55].

For the source space activations, ROIs were based on the standard 68 brain region Desikan-Killiany atlas [63] using the Colin-27 head model [64]. Artifacts still remaining in source space within individual subject data were removed using the Grubbs statistical test applied iteratively using spline interpolation - an option available within the MATLAB isoutlier function [51], and population outliers across all sessions and subjects source data were removed using the >5SD criterion. ROIs were further grouped into canonical cognitive control networks that can undergo task-dependent modulation, i.e. the fronto-parietal network (FPN), cingulo-opercular network (CON) and the default mode network (DMN) [65-68]; ROIs in each network are shown in Table S1.

**Table S1.**

| Network | Regions of Interest | ROI index in DK Atlas |
| --- | --- | --- |
| FPN | caudalmiddlefrontal L | 5 |
| FPN | caudalmiddlefrontal R | 6 |
| FPN | rostralmiddlefrontal L | 55 |
| FPN | rostralmiddlefrontal R | 56 |
| FPN | superiorparietal L | 59 |
| FPN | superiorparietal R | 60 |
| CON | caudalanteriorcingulate L | 3 |
| CON | caudalanteriorcingulate R | 4 |
| CON | insula L | 19 |
| CON | insula R | 20 |
| CON | parsopercularis L | 37 |
| CON | parsopercularis R | 38 |
| CON | parsorbitalis L | 39 |
| CON | parsorbitalis R | 40 |
| CON | parstriangularis L | 41 |
| CON | parstriangularis R | 42 |
| CON | superiorfrontal L | 57 |
| CON | superiorfrontal R | 58 |
| CON | transversetemporal L | 67 |
| CON | transversetemporal R | 68 |
| DMN (aDMN) | frontalpole L | 11 |
| DMN (aDMN) | frontalpole R | 12 |
| DMN (aDMN) | lateralorbitofrontal L | 25 |
| DMN (aDMN) | lateralorbitofrontal R | 26 |
| DMN (aDMN) | medialorbitofrontal L | 29 |
| DMN (aDMN) | medialorbitofrontal R | 30 |
| DMN (aDMN) | rostralanteriorcingulate L | 53 |
| DMN (aDMN) | rostralanteriorcingulate R | 54 |
| DMN (pDMN) | inferiorparietal L | 15 |
| DMN (pDMN) | inferiorparietal R | 16 |
| DMN (pDMN) | isthmuscingulate L | 21 |
| DMN (pDMN) | isthmuscingulate R | 22 |
| DMN (pDMN) | precuneus L | 51 |
| DMN (pDMN) | precuneus R | 52 |
| DMN (mtlDMN) | entorhinal L | 9 |
| DMN (mtlDMN) | entorhinal R | 10 |
| DMN (mtlDMN) | inferiortemporal L | 17 |
| DMN (mtlDMN) | inferiortemporal R | 18 |
| DMN (mtlDMN) | middletemporal L | 31 |
| DMN (mtlDMN) | middletemporal R | 32 |
| DMN (mtlDMN) | parahippocampal L | 35 |
| DMN (mtlDMN) | parahippocampal R | 36 |
| DMN (mtlDMN) | superiortemporal L | 61 |
| DMN (mtlDMN) | superiortemporal R | 62 |
| DMN (mtlDMN) | temporalpole L | 65 |
| DMN (mtlDMN) | temporalpole R | 66 |

Regions of Interest (ROIs) grouped within the three cognitive control networks are enumerated for the fronto-parietal network (FPN), cingulo-opercular network (CON) and the default mode network (DMN). The DMN includes the anterior DMN (aDMN), posterior DMN (pDMN) and the medial temporal DMN (mtlDMN). Corresponding indices of ROIs within the Desikan-Killiany (DK) atlas are also shown. L: left, R: right.
